# A green approach to Azo dye C.I. Disperse blue 291 treatment: mitigating ecotoxicological effects through enzymatic and adsorptive strategies using endophytic fungi

**DOI:** 10.1007/s11274-026-04841-0

**Published:** 2026-02-23

**Authors:** Esperança Edna Alexandre Chibite, Débora Elisa Antunes de Mendonça, Mariane Aparecida Franco de Godoy, Adriane do Nascimento, João Artur dos Santos Oliveira, Brian Alvarez Ribeiro de Melo, Michele Cristina Heck, Julio Cesar Polonio, Igor Vivian Almeida, Veronica Elisa Pimenta Vicentini

**Affiliations:** 1 Faculty of Science and Technology, Licungo University of Mozambique, Av. Julius Nyerere, Quelimane, Zambézia 1621 Mozambique; 2 Department of Biotechnology, Genetics, and Cell Biology, State University of Maringá, Av. Colombo, 5790, Jardim Universitário, Maringá, Paraná 87020-900 Brazil; 3 Department of Physics, State University of Maringá, Av. Colombo, Jardim Universitário, Maringá, Paraná 5790, 87020-900 Brazil; 4 Department of Statistics, State University of Maringá, Av. Colombo, Jardim Universitário, Maringá, Paraná 5790, 87020-900 Brazil; 5 Federal Rural University of Amazonia, Rua Professora Antônia Cunha de Oliveira, S/N, Vila Nova, Capitão Poço, Pará, 68650-000 Brazil

**Keywords:** *Alternaria alternata*, *Artemia salina*, *Aspergillus flavus*, Azo dyes, Biodegradation, Biosorption

## Abstract

Textile dyes are persistent pollutants that pose significant risks to the environment and living organisms. Before being released into aquatic ecosystems, these dyes require effective treatment. Among these dyes, the azo dye Disperse Blue 291 exhibits relevant toxicological and mutagenic activity. However, a scarcity of studies has been identified that evaluate the effects of the DB291 dye before and after decolorization. Endophytic fungi have shown promising potential in bioremediation, particularly through biosorption and biodegradation. This study aimed to evaluate the ability of endophytic fungi to decolorize the textile dye Disperse Blue 291 (DB291). Seven fungal strains were screened in a minimal medium containing 10 µg/mL of DB291 for seven days. *Aspergillus flavus* CL07 and *Alternaria alternata* PL75 showed decolorization rates above 90% and were selected for further analysis. Both fungi achieved complete decolorization, *A. flavus* CL07 within 48 h and *A. alternata* PL75 within 168 h. Spectroscopic and enzymatic analyses suggested that biosorption was the main mechanism for *A. flavus* CL07, while *A. alternata* PL75 also employed extracellular laccase activity in dye degradation. Infrared spectroscopy analysis indicated chemical interactions between dye molecules and fungal biomass. Ecotoxicity assays using *Artemia salina* revealed that the original dye was highly toxic, causing developmental arrest and complete lethality. In contrast, the decolorization product from *A. alternata* PL75 exhibited no significant toxicity, supporting normal development in all tested stages. These results suggest that endophytic fungi, particularly *A. alternata* PL75, offer an effective and environmentally safe approach to the bioremediation of textile dye-contaminated environments.

**Graphical abstract:**

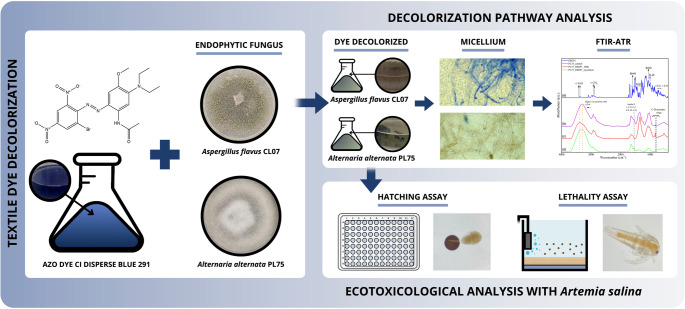

## Introduction

Synthetic dyes represent up to 75% of the global dye market. Among them, azo dyes, characterized by the presence of –N = N– bonds, constitute the largest class of synthetic aromatic dyes. These compounds are widely utilized across various industries, including textiles, cosmetics, food, pharmaceuticals, and paper manufacturing (Qin et al. 2024).

The textile industry, one of the most prominent sectors in the global economy, is identified as one of the leading polluters of water resources. According to global estimates, more than 200,000 tons of effluents enriched with harmful substances are discharged annually by textile industries (Jegatheesan et al. 2021; Chau et al. 2023). When these effluents reach the environment, they become recalcitrant, exposing humans to significant health risks and causing adverse effects on the local biota (Tsuboy et al. 2007; Vilchis‑Carmona et al. 2021).

The azo dye C.I. Disperse Blue 291 (DB291) (2-(2-bromo-4,6-dinitrophenyl)azo-5(diethylamino)−4-methoxyacetanilide) is a hydrophobic dye used for coloring various fibers, particularly polyester and polyamide, and is therefore widely utilized in the textile industry worldwide. This dye is a precursor to one of the compounds belonging to the 2-phenylbenzotriazole class (PBTA-7), a potent mutagen that has already been detected in waters receiving discharges from textile industries (Watanabe et al. 2002; Fernandes et al. 2019).

The mutagenicity of this dye has been confirmed in in vitro tests using the Salmonella/microsome assay, which revealed the compound’s ability to cause frame-shift mutations and base pair substitutions (Umbuzeiro et al. 2005). In liver tumor cell lines, comet assay, micronucleus assay, and cell viability tests revealed an increase in comet tail length and the frequency of micronuclei, along with a decrease in cell viability (Tsuboy et al. 2007). In vivo tests on mouse bone marrow cells indicated that the dye has mutagenic effects, but without a dose-response relationship. Additionally, no primary DNA damage or changes in gene expression were observed (Fernandes et al. 2019).

Vacchi et al. (2017) reported that DB291 (0.05 µg/L) and several other dyes were detected in river waters used for human consumption. It is important to emphasize that there is a scarcity of studies reporting the biodegradation of this dye. However, due to the structural complexity of azo dyes combined with their recalcitrance, the discharge of these dyes and their effluents into water bodies is concerning, highlighting the need for prior treatment. Chemical treatments have proven inefficient, as they not only entail high costs but also generate by-products that may be more toxic than the original compound, thereby introducing additional environmental challenges (Kadam et al. 2011; Chau et al. 2023).

In this context, biological treatment has gained increasing attention due to its efficiency, cost-effectiveness, and environmentally friendly nature (Jadhav and Govindwar 2006; Durruty et al. 2015; Dayi et al. 2018; Alam et al. 2023). Notably, there are few studies focused on the decolorization of the DB291 dye. However, there is extensive literature reporting the decolorization of textile dyes using fungi isolated from contaminated sites. Particular emphasis has been placed on endophytic fungi, which, in addition to serving as a natural source of biomolecules with significant biotechnological potential (Pushparaj et al. 2023), possess cell membranes with unique physical and chemical properties, along with ligninolytic enzymes they secrete. This combination equips them with exceptional capabilities for biosorption and biodegradation of pollutants, especially when compared to bacteria (Chau et al. 2023). They also possess a strong adaptability to regulate their metabolic pathways under different environmental conditions (Yan et al. 2023), making them strong candidates for the decolorization and biodegradation of azo dyes.

Recent studies report the efficiency of endophytic fungi in the decolorization of various textile dyes. Yan et al. (2023) investigated the decolorization of Congo Red, Eriochrome Black T, and Neutral Red dyes using the fungus *Penicillium janthinellum* LM5 and achieved decolorization rates of 99%, 98%, and 96.5% within 48 h, respectively. Polli et al. (2023) evaluated the decolorization of the Reactive Black 5 dye using the fungus *Aspergillus flavus* CL07 and achieved a decolorization rate of 95.1% within seven days, with this fungus demonstrating tolerance to all tested concentrations.

According to Brack et al. (2019), dyes represent a complex mixture, and simple monitoring provides limited insight into the potential ecotoxicological impacts they may have on aquatic ecosystems. Consequently, a comprehensive approach to assessing the effectiveness of treatments for dyes and textile effluents involves conducting toxicological analyses with a variety of test organisms. Among these, *Artemia salina*, a microcrustacean commonly utilized in ecotoxicological studies for initial toxicity screening of xenobiotics, is particularly notable.

Given the scarcity of research on the azo dye DB291, a hydrophobic, non-ionic, and dispersed compound characterized by its environmental persistence and recalcitrance and the lack of studies evaluating its toxicological potential or that of its decolorization products, this research aimed to identify endophytic fungi capable of decolorizing this dye. Following the selection of the fungi, the enzymes involved in the decolorization process were analyzed, along with the rate of decolorization. Potential decolorization pathways were investigated through spectrophotometric analyses. Additionally, a toxicological assessment of the decolorized product was performed using the bioindicator *A. salina*, with the aim of evaluating the ecological safety of the process. The novelty of this study lies in the use of endophytic fungi for the treatment of this dye and in the ecotoxicological evaluation of both the dye and its decolorization product. This represents a notable advance in the sustainable decolorization of textile dyes and their effluents. Moreover, it constitutes a promising application for the bioremediation of environments contaminated with textile dyes.

## Materials and methods

### Azo dye

**Fig. 1:**
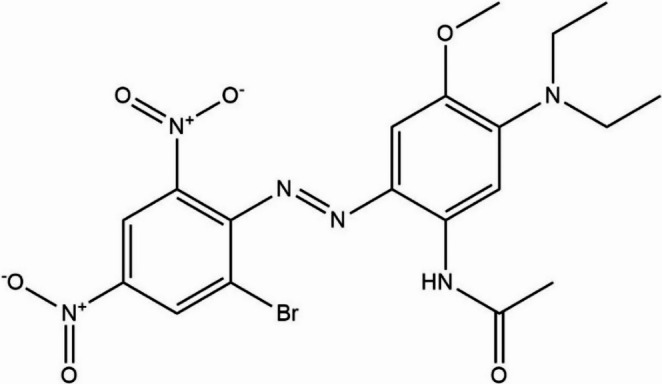
Chemical structure of azo dye C.I. Disperse Blue 291

The azo dye used in this study was C.I. Disperse Blue 291 (2-[(2-bromo-4,6-dinitrophenyl)azo]−5(diethylamino)−4-methoxyacetanilide) (LGC DR EHRENSTORFERTM, CAS 56548-64−2, 88.4% (g/g) purity). The dye has the molecular formula C_19_H_21_BrN_6_O_6_ and a molecular weight of 509.31 g/mol (Fig. 1).

### Endophytic fungi

Azo dyes exhibit high toxicological potential for both humans and the environment. Considering that endophytic fungi and their metabolites possess significant biodegradation and bioremediation capabilities for effluents contaminated with these dyes, the hypothesis of this study is that, after selecting the fungi most effective in decolorizing the azo dye DB291, the products resulting from fungal decolorization will not exhibit toxicological potential when assessed using hatching and aquatic toxicity assays with *Artemia salina*, thereby enabling the reuse of water generated from this decontamination process.

Seven endophytic fungi were selected from the Collection of Endophytic Microorganisms and the Environment (CMEA) at the Microbial Biotechnology Laboratory (LBIOMIC) of the State University of Maringá (UEM) (Table 1), with registration number A5095ED in the National System for the Management of Genetic Heritage and Associated Traditional Knowledge (SisGen, Brazil). These fungal strains exhibit significant biotechnological potential and have been used at LBIOMIC for various research projects, yielding satisfactory results. They constituted the initial phase of this exploratory study, which aimed to identify fungi with the potential to decolorize DB291. For reactivation, a mycelial disc from each strain was transferred to Potato Dextrose Agar (PDA, pH 6.8) and incubated at 28 °C for seven days.

**Table 1:** Endophytic fungi from the collection of endophytic microorganisms and the environment (CMEA/LBIOMIC) and their respective host plants

Endophytic fungi	Strain code	Host plant	Reference
*Fusarium circinatum*	Tg134	*Tibouchina granulosa* (Myrtales: Melastomataceae)	Golias et al. 2020
*Fusarium citri*	SL09	*Serjania laruoteana* (Sapindales: Sapindaceae)	Ribeiro et al. 2021
*Alternaria alternata*	PL75	*Pachystachys lutea* (Lamiales: Acanthaceae)	Ribeiro et al. 2018
*Colletotrichum gloeosporioides*	JB158	*Justicia brandegeana* (Lamiales: Acanthaceae)	Silva et al. 2020
*Aspergillus flavus*	CL07	*Chromolaena laevigata* (Asterales: Asteraceae)	Balbinot et al. 2021
*Paecilomyces formosus*	IG03	*Helianthus annuus* (Asterales: Asteraceae)	Bulla et al. 2017
*Lasiodiplodia* sp.	G20-20	*Piper hispidum* (Piperales: Piperaceae)	Orlandelli et al. 2012

### Decolorization assay

The selection of endophytic fungi for the decolorization of the DB291 dye was conducted according to Bulla et al. (2017), with modifications. After pre-cultivation of the endophytes on PDA medium (pH 6.8) for seven days at 28 °C, a 6-mm-diameter disc from each fungal colony was transferred to minimal culture medium (MM) (6.0 g/L NaNO_3_, 5.0 g/L KCl, 1.5 g/L KH_2_PO_4_, 0.5 g/L MgSO_4_·7H_2_O, 0.001 g/L ZnSO_4_, 0.001 g/L FeSO_4_, 10.0 g/L glucose, and 1,000 mL of distilled water; pH 6.8).

Due to its low water solubility, the DB291 dye was diluted in 0.1% DMSO and 0.1% Tween 20, and added to the MM to achieve a final concentration of 10 µg/mL. This concentration, which was established as the maximum dilution level in preliminary tests, is 200,000-fold higher than those typically found in the environment (0.05 µg/L; Vacchi et al. 2017). The treatment groups consisted of the following fungi: *Fusarium circinatum* Tg134; *Fusarium citri* SL09; *Alternaria alternata* PL75; *Colletotrichum gloeosporioides* JB158; *Aspergillus flavus* CL07; *Paecilomyces formosus* IG03; and *Lasiodiplodia* sp. G20-20. Erlenmeyer flasks (100 mL) containing 50 mL of MM + DB291 were individually inoculated with a 6-mm diameter disc of each fungal strain. Two uninoculated control groups were used: one containing only MM and the other containing MM + DB291 dye. The entire experiment was conducted in triplicate, and all Erlenmeyer flasks were incubated under orbital shaking (150 rpm) at 28 °C for seven days.

Absorbance measurements were recorded on the fifth and seventh days of cultivation to determine the percentage of dye decolorization. Prior to the measurements, a spectral scanning analysis of the dye diluted in MM was conducted using a Biochrom spectrophotometer (LIBRA S60PC) within the wavelength range of 300 to 900 nm to identify the optimal absorption peak.

Following this, the samples were filtered, and 1 mL aliquots of the supernatant were transferred to polystyrene optical cuvettes. Absorbance readings were then taken at 600 nm using the spectrophotometer. The decolorization percentage (D%) was calculated using the following formula: D%=(A_0_-A_t_/A_0_) × 100, where A_0_ represents the absorbance value of the controls and Aₜ represents the absorbance value of the treatments. The absorbance data obtained were statistically analyzed using the Sisvar software through analysis of variance (ANOVA), and the means of the treatment absorbances were compared using the Scott-Knott test at a 5% significance level. The Scott-Knott post-hoc test was employed to avoid overlapping groups, thereby enhancing the discriminatory power to identify the most effective treatments.

### Decolorization rate (V_d_)

The decolorization rate (V_**d**_) analysis was performed only with the strains that stood out statistically in the initial decolorization assay. The preparation of the inoculum, treatment, and incubation were carried out as described in the previous section. Absorbance readings were taken daily for seven days. Aliquots of 1 mL were measured using a spectrophotometer at a wavelength of 600 nm. For the calculation of V_**d**_, the following formula was used: V_d_ = dD/d_t_, where V_d_ represents the decolorization rate and dD/d_t_ is the derivative of the decolorization percentage over time.

### Quantification of residual dye in the medium and adhered to the mycelium

The quantification of residual dye in the culture medium after decolorization was estimated by constructing a standard curve with different dye concentrations. The dye was diluted as previously described (in the Decolorization Assay) in final concentrations of 0, 1, 2, 4, 6, 8, 10, and 12 µg/mL in MM. To determine the amount of dye adhered to the fungus, after filtering the fungal decolorization product, the mycelium was macerated to extract the dye adhered to it. A liquid-liquid extraction of the dye was performed using ethyl acetate as the organic solvent to facilitate phase partitioning. A standard curve was constructed by diluting the dye in ethyl acetate at the concentrations described earlier. Absorbance readings were taken at a wavelength of 600 nm, all in triplicate.

### Morphology of fungal biomass in the presence of DB291 dye

To better understand the decolorization mechanisms of the dye, visual and microscopic observations were made of the mycelia of *A. flavus* CL07 and *A. alternata* PL75. Mycelial samples from the control and treatment groups of both fungi, after decolorization (after five and seven days, respectively), were collected, placed on a microscope slide, covered with a coverslip, and examined under a light microscope. Photographic records were made using a light microscope (LEICA ICC50W). Visual observation of the biomass was performed through photographic recording.

### Enzymatic analysis of the decolorization products

The total enzymatic activity, laccase activity, and peroxidase-like activity (estimated by the difference between total ABTS-oxidizing activity and laccase activity) were evaluated by monitoring the oxidation of ABTS (2,2-azino-bis(3-ethylbenzothiazoline-6-sulfonic acid) using a spectrophotometer (Spectra MAX M2, Molecular Devices) at 420 nm, with 30-second intervals over a period of 20 min, following the methodology described by Machado and Matheus (2006), with modifications. To calculate the enzymatic activity, it was considered that one enzyme unit (U) corresponds to the amount of enzyme capable of oxidizing 1 µL of substrate per minute, using the molar extinction coefficient for oxidized ABTS (ε = 36,000 M⁻¹ cm⁻¹).

The enzymatic activity was determined according to the following formula: U/L=(A)x(V_t_)x(D_f_)x(10^6^)/(t)x(ε)x(d)x(V_s_) (Baltiera-Trejo et al. 2015), where A is the absorbance measured at the midpoint of the linear phase, V_t_ is the total reaction volume, D_f_ is the dilution factor (1:10), 10^6^ is a correction factor, t is the time for absorbance measurement, ε is the molar extinction coefficient (36,000 M⁻¹ cm⁻¹), d is the optical path length (1 cm), and V_s_ is the sample volume.

The analysis of enzymatic activity results was performed by comparing the means using the Tukey test, with the assistance of the statistical program GraphPad InStat (version 3.02). The significance level was set at p ˂ 0.05.

Total enzymatic activity was determined according to the methodology described by Machado and Matheus (2006), with modifications. Solutions were prepared containing 1.2 mL of enzymatic extract (supernatant resulting from fungal cultivation), 0.5 mL of citrate-phosphate buffer (pH 4), 0.1 mL of 2 mM hydrogen peroxide, and 0.2 mL of 5 mM ABTS. Laccase activity was determined by the oxidation of ABTS (as described previously), in the absence of hydrogen peroxide, using bovine catalase (0.07 U) for a 15-minute pre-incubation. Peroxidase-like activity was calculated by subtracting the laccase activity from the total ABTS oxidation activity.

### Fourier transform infrared spectroscopy with attenuated total reflectance

The decolorization of the DB291 dye was analyzed using Fourier Transform Infrared Spectroscopy (FTIR), conducted on an FTIR spectrometer equipped with an Attenuated Total Reflectance (ATR) accessory (Bruker, model Vertex 70, Germany) at the Center for Advanced Materials Analysis (CAM) of the Research Support Complex (COMCAP/UEM). The characterization of the DB291 dye and the decolorization products samples from *A. flavus* CL07 (after 48 h of decolorization) and *A. alternata* PL75 (after 168 h of decolorization), as well as the fungal biomasses, was performed and compared with the respective controls. Before the analyses, the samples were lyophilized. The spectral range used was between 4,000 and 400 cm⁻¹ in each spectrum, with an average of 128 scans and a resolution of 4 cm⁻¹.

### Ecotoxicology assessment

#### *Artemia salina* hatching assay

The hatching assay of *A. salina* cysts was performed according to Wang et al. (2017), with modifications. The hatching phases were described following Carballo et al. (2002) and Rowarth and Macrae (2018). The cysts, obtained from a local commercial source, were hydrated in distilled water at approximately 25 °C for two hours. A total of 880 cysts were divided into a 96-well plate, with 10 cysts in each well, and 200 µL of treatment solution for each group.

A total of 11 groups were analyzed, with 8 replicates each: Negative Control (NC), Positive Control (PC), Minimal Medium (MM), Minimal Medium and Solvent (MMS), Water and Solvent (WS), *Alternaria alternata* PL75 (Aa), *Aspergillus flavus* CL07 (Af), Disperse Blue 291 2 µg/mL (DB2), Disperse Blue 291 10 µg/mL (DB10), Decolorization product of *Alternaria alternata* PL75 + DB291 10 µg/mL (PDAa), and Decolorization product of *Aspergillus flavus* CL07 + DB291 10 µg/mL (PDAf).

The negative and positive control groups were composed of saline water (35 g/L of marine salt in distilled water) and saline water with acetaminophen (800 µg/mL), respectively. All other groups were supplemented with marine salt (35 g/L). The experiment was conducted in a shaker incubator at 26 °C ± 2, 100 rpm, under continuous illumination. Four observations were made under a stereomicroscope at 6, 12, 24, and 48 h to record the developmental stages of the microcrustacean, followed by photographic documentation.

#### *Artemia salina* lethality assay

This assay was performed according to Meyer et al. (1982), with modifications. The *A. salina* cysts were placed to hatch in an aquarium containing saline water (35 g/L of marine salt in autoclaved distilled water) at approximately 25 °C, under continuous illumination and aeration for 48 h. After this period, 880 nauplii were placed into a 96-well plate, with 10 nauplii per well and a volume of 200 µL of the treatment solution. Eleven groups were analyzed, as described in Sect. 2.9.1. The plates were placed in a shaker incubator at 26 °C ± 2, 100 rpm, under continuous illumination. Observations were made under a stereomicroscope at 24 and 48 h of exposure to the treatments to determine the number of live and dead nauplii. Nauplii were considered dead if they did not move during observation, even when the plate was gently shaken.

The hatching and lethality data were analyzed using the R statistical software (R Core Team 2021). A generalized linear model with a binomial response and a logit link function (logistic regression) was employed, followed by Tukey’s multiple comparisons performed with the multcomp package (Hothorn et al. 2008). A significance level of 5% was considered for all analyses.

## Results

### Selection of endophytic fungi

**Fig. 2:**
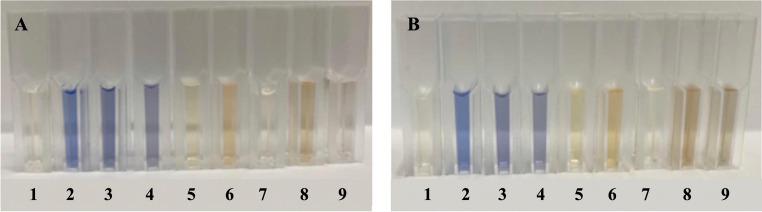
Aliquots (1mL) on the 5th (**A**) and 7th (**B**) days of the DB291 dye decolorization assay. 1: MM control; 2: MM + DB291 (10 µg/mL) control; 3: *F. circinatum* Tg134; 4: *F. citri* SL09; 5: *A. alternata* PL75; 6: *C. gloeosporioides* JB158; 7: *A. flavus* CL07; 8: *P. formosus* IG03; 9: *Lasiodiplodia* sp. G20-20

**Fig. 3:**
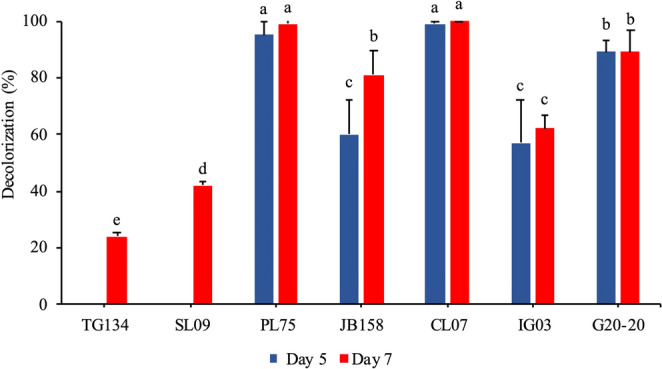
Percentage of decolorization (%D) of the fungi: *F. circinatum* TG134; *F. citri* SL09; *A. alternata* PL75; *C. gloeosporioides* JB158; *A. flavus* CL07; *P. formosus* IG03; *Lasiodiplodia* sp. G20-20, after the 5th and 7th days of cultivation ^a, b, c, d, e^ Statistically significant differences between the treatments

As shown in Figs. 2 and 3, all the fungi selected for the initial screening exhibited some degree of decolorization activity against the azo textile dye DB291. However, the *A. flavus* CL07 and *A. alternata* PL75 strains demonstrated the most promising decolorization capabilities, with decolorization percentages of 100% and 99%, respectively, after the seventh day of cultivation. Thus, these two fungi were chosen for further monitoring of the decolorization time and speed (V_d_), the enzymes produced, possible decolorization pathways, and to obtain the decolorization products for subsequent ecotoxicological assessment.

### Decolorization rate (V_d_) and residual dye analysis

**Fig. 4:**
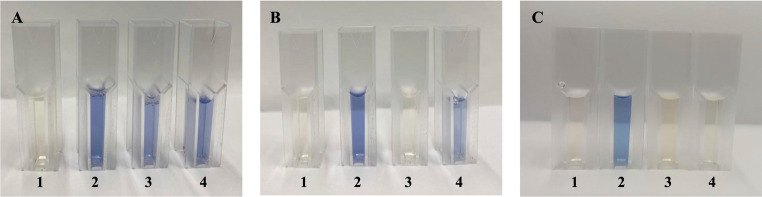
Decolorization of the DB291 dye by endophytic fungi. **A**: 24 h; **B**: 120 h; and **C**: 168 h; 1: MM Control; 2: MM + DB291 (10 µg/mL) control; 3: *A. flavus* CL07; 4: *A. alternata* PL75

**Fig. 5:**
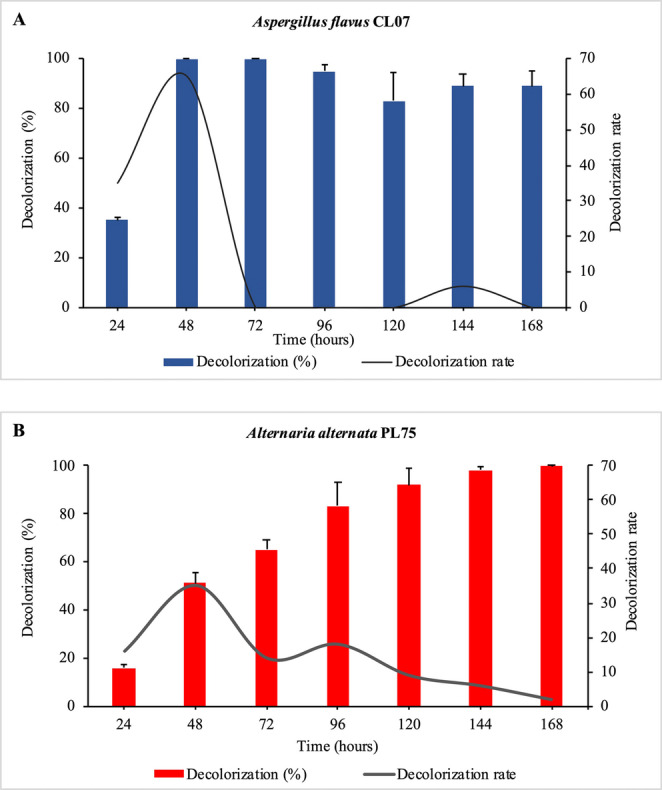
Percentage and decolorization rate of *Aspergillus flavus* CL07 (**A**) and *Alternaria alternata* PL75 (**B**). Decolorization values are expressed as mean ± standard deviation

Figure 4 qualitatively illustrates the color changes of the treatments throughout the decolorization process. The two endophytic fungal strains, *A. flavus* CL07 and *A. alternata* PL75, exhibited distinct behaviors in terms of decolorization rate (V_d_), as shown in Fig. 5. Both strains showed the highest decolorization rate within the first 48 h. The *A. flavus* CL07 strain reached its maximum decolorization rate of 65% per day, while *A. alternata* PL75 achieved 35% per day. Complete decolorization of the DB291 dye was observed at different times for each strain: *A. flavus* CL07 reached the maximum decolorization rate of 100% in 48 and 72 h, while *A. alternata* PL75 only achieved complete decolorization after 168 h. These results highlight the efficiency of the *A. flavus* CL07 strain, which exhibited faster decolorization, achieving total removal of the dye in less time compared to *A. alternata* PL75.

**Fig. 6:**
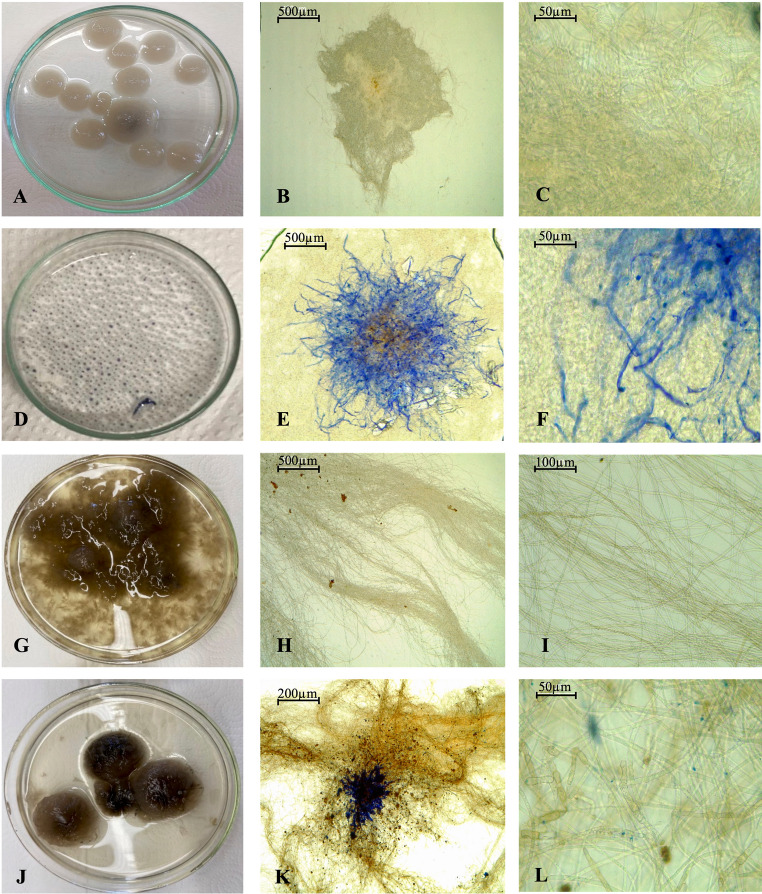
Mycelium of the fungi after 5 and 7 days of cultivation for *A. flavus* CL07 and *A. alternata* PL75, respectively. **A**, **B**, and **C**: *A. flavus* CL07 without dye; **D**, **E**, and **F**: *A. flavus* CL07 with DB291 dye; **G**, **H**, and **I**: *A. alternata* PL75 without dye; **J**, **K**, and **L**: *A. alternata* PL75 with DB291 dye

Analysis revealed that both fungi achieved their highest decolorization rates on the second day. *A. flavus* CL07 exhibited fluctuations after the stability observed at 48 and 72 h, with 100% decolorization (Fig. 6A), while *A. alternata* PL75 displayed a decreasing trend over the seven days of decolorization (Fig. 6B).

Through UV-Vis spectrophotometry, the average residual concentration of the dye in the culture medium was estimated after the fifth day of cultivation for the *A. flavus* CL07 strain and the seventh day for *A. alternata* PL75. The results indicate a final concentration of approximately 1 to 2 µg/mL of the dye in the culture medium after treatment with both fungi, representing a reduction of about 80% compared to the initial concentration used (10 µg/mL), with a positive correlation (R² = 0.9922).

It is also worth noting that during the decolorization process, the fungal biomass exhibited an atypical coloration for these fungi (Fig. 6), with bluish tones around the mycelium for *A. flavus* CL07 and darker shades for *A. alternata* PL75. This suggests that the dye became impregnated in the mycelial walls of these fungi, indicating a mechanism employed by these fungi for dye removal.

### Enzyme activity analysis

Table 2 shows the average enzyme activity produced by *A. flavus* CL07 and *A. alternata* PL75 on the fifth and seventh days of decolorization. According to the presented data, *A. flavus* CL07 exhibited enzyme activity values below 1 U/mL, with statistically significant differences compared to its control. On the other hand, *A. alternata* PL75 recorded higher values, with total ABTS-oxidizing activity at 5.26 U/mL ± 1.86, laccase at 3.85 U/mL ± 1.32, and peroxidase-like activity at 1.41 U/mL ± 0.60, though there were no statistically significant differences compared to its control.

**Table 2:** Enzymatic activities of *Aspergillus flavus* CL07 and *Alternaria alternata* PL75 strains and their products of decolorization of disperse blue 291 dye. The values represent the mean and standard error

Fungi	Enzyme (U/mL)	Control	Product of decolorization
***Aspergillus flavus*** CL07	ABTS	0.54 ± 0.01	0.98^*^ ± 0.05
	Laccase	0.54 ± 0.03	0.07^*^ ± 0.03
	Peroxidase-like activity	0.00 ± 0.01	0.91^*^ ± 0.03
***Alternaria alternata*** PL75	ABTS	3.42 ± 1.44	5.26 ± 1.86
	Laccase	2.97 ± 1.33	3.85 ± 1.32
	Peroxidase-like activity	0.46 ± 0.18	1.41 ± 0.60

^*^Statistically significant values (p˂ 0.05)

**Fig. 7:**
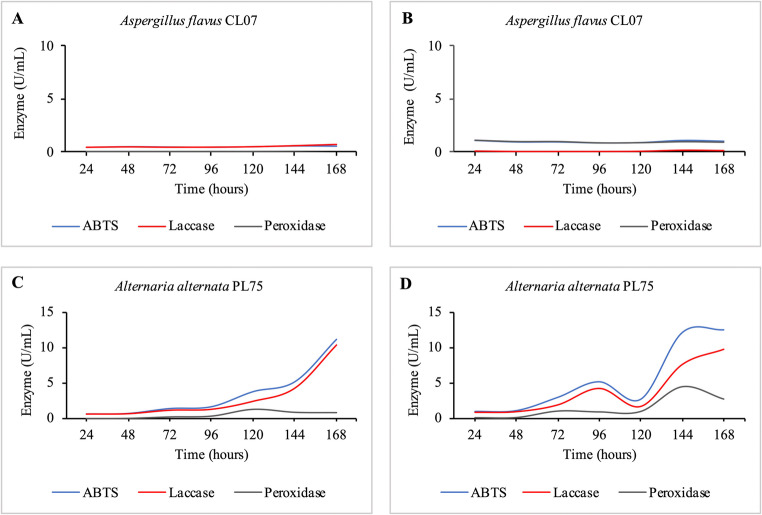
Enzymes ABTS, Laccase, and Peroxidase-like activity produced by the fungi in Enzyme Units (U/mL) during the decolorization process. **A**: *Aspergillus flavus* CL07 control group; **B**: *Aspergillus flavus* CL07 treatment group; **C**: *Alternaria alternata* PL75 control group; **D**: *Alternaria alternata* PL75 treatment group

Figure 7 shows the enzyme activity recorded over the seven days of the experiment, which indicates that *A. flavus* CL07 exhibited some activity at 24 h (0.07 U/mL), but no activity was detected at 48, 72, and 96 h. *A. alternata* PL75 (Fig. 7D) demonstrated an increase in enzymatic activity until 96 h, with ABTS at 5.06 U/mL and laccase at 4.22 U/mL, followed by a fluctuation at 120 h. However, a decrease in activity was recorded, with ABTS at 2.62 U/mL, laccase at 1.70 U/mL, and peroxidase-like activity at 0.90 U/mL. At 144 h, the enzymatic activity increased significantly, reaching 12 U/mL for ABTS, 7.58 U/mL for laccase, and 4.42 U/mL for peroxidase-like. The maximum laccase activity produced by *A. flavus* CL07 and *A. alternata* PL75 was 0.21 U/mL (144 h) and 9.72 U/mL (168 h), respectively.

### Dye degradation based on UV-Vis and FTIR-ATR

Based on the UV-Vis spectrophotometry results for DB291 following fungal mycelium maceration, together with the FTIR-ATR spectrum analysis, the potential decolorization pathways of this dye by the evaluated fungi can be elucidated. The maceration results demonstrated the presence of the dye at concentrations of 10 µg/mL for *A. flavus* CL07 and 5 µg/mL for *A. alternata* PL75, exhibiting a strong positive correlation (R² = 0.998).

**Fig. 8:**
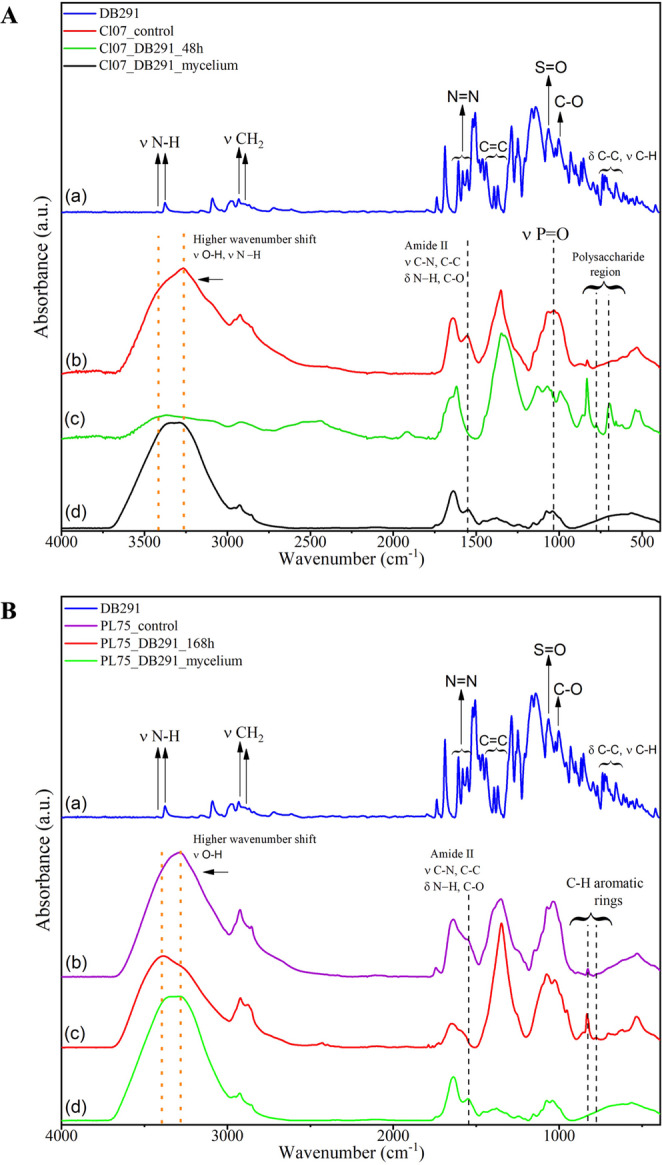
Infrared Absorption Spectra by Attenuated Total Reflectance of samples. **A**: *A. flavus* CL07; (a): DB291 dye; (b): CL07 control; (c): CL07 DB291 after 48 h treatment; (d): mycelium after decolorization; **B**: *A. alternata* PL75; (a): DB291 dye; (b): PL75 control; (c): PL75 DB291 treatment after 168 h; (d): mycelium after decolorization

Figure 8A presents the FTIR-ATR spectra of the DB291 dye (a), the control *A. flavus* CL07 fungus (b), post-decolorization (c), and the fungal mycelium (d). Analysis of these spectra reveals the disappearance of certain bands after decolorization that were present in the control fungus spectrum (b), notably at 1550 cm⁻¹ (amide II) and 1030 cm⁻¹ (P = O). Additionally, a shift was observed in the spectral region around 3267 cm⁻¹, corresponding to N–H bonds of amides and O–H groups of carboxylic acids, accompanied by the emergence of two new bands at 776 cm⁻¹ and 695 cm⁻¹.

Regarding the spectrum of *A. alternata* PL75 (Fig. 8B), a shift in the band within the 3700–3000 cm⁻¹ region (associated with O-H bonds) was evident throughout the decolorization treatment. In the fingerprint region (900–700 cm⁻¹), two bands of lower intensity, attributed to C–H bonds, were identified in the control group. However, following decolorization, an increase in intensity occurred in this region, which subsequently disappeared in the spectrum of the mycelium after decolorization.

These spectral modifications indicate biochemical transformations in the dye molecules mediated by fungal activity, shedding light on the mechanisms underlying dye degradation and removal by the fungal strains studied.

### Ecotoxicology of the DB291 dye using the *Artemia salina* bioassay

#### Hatching assay

**Fig. 9:**
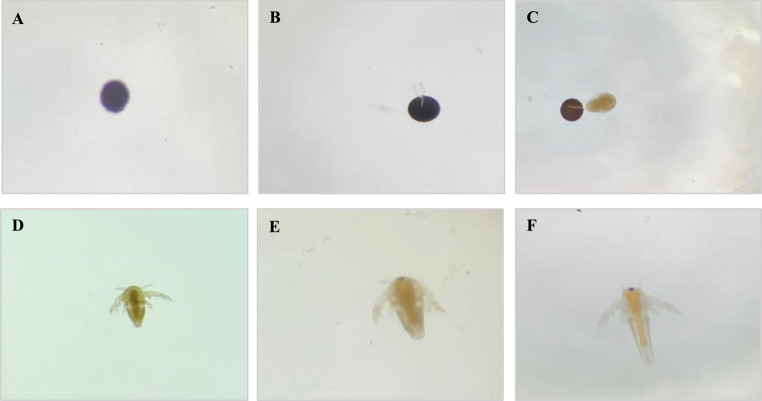
Developmental stages of *Artemia salina*

The hatching rate of *A. salina* was recorded at various stages: **A**, cyst; **B**, decapsulating cyst; **C**, nauplius emerging; **D**, nauplius instar I; **E**, nauplius instar II; and **F**, nauplius instar III (Fig. 9).

**Fig. 10:**
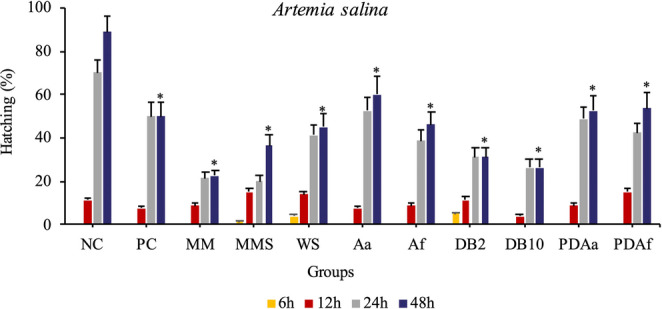
Hatching percentage of cysts after 6, 12, 24, and 48 h of exposure. NC: Negative Control; PC: Positive Control; MM: Minimal Medium; MMS: Minimal Medium and Solvent; WS: Water and Solvent; Aa: *A. alternata* PL75; Af: *A. flavus* CL07; DB2: DB291 dye 2 µg/mL; DB10: DB291 dye 10 µg/mL; PDAa: fungal decolorization product of *A. alternata* PL75; PDAf: fungal decolorization product of *A. flavus* CL07. Bars represent hatching percentages and standard error *Statistically significant difference compared to the negative control (p˂0.05)

Figure 10 shows the hatching percentages of the cysts at 6, 12, 24, and 48 h of exposure. According to these data, hatching and nauplius development were clearly influenced by the treatment, dye concentration, and exposure duration. After 48 h of exposure, only the groups NC, Aa, PDAa, and PDAf exhibited hatching rates above 50%.

**Fig. 11:**
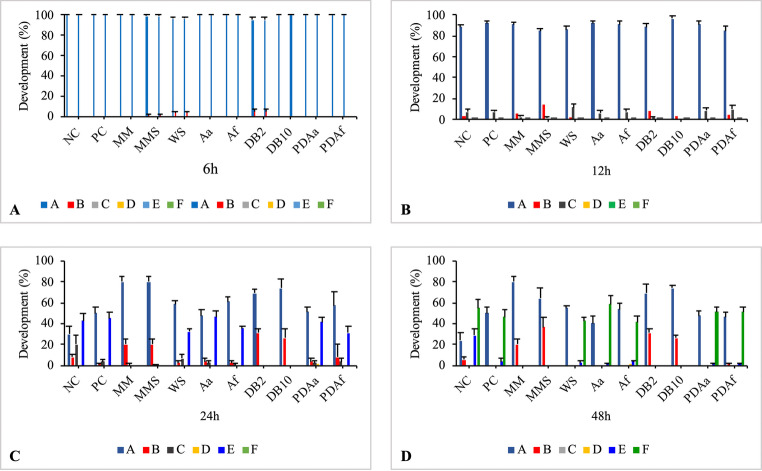
Developmental stages of *Artemia salina* cysts after 6, 12, 24, and 48 h of exposure. **A**: Cyst; **B**: Cyst de-capsulating; **C**: Nauplius emerging; **D**: Nauplius at Instar I; **E**: Nauplius at Instar II; **F**: Nauplius at Instar III. Groups: NC: Negative Control; PC: Positive Control; MM: Minimal Medium; MMS: Minimal Medium and Solvent; WS: Water and Solvent; Aa: *A. alternata* PL75; Af: *A. flavus* CL07; DB2: DB291 dye 2 µg/mL; DB10: DB291 dye 10 µg/mL; PDAa: Fungal decolorization product of *A. alternata* PL75; PDAf: Fungal decolorization product of *A. flavus* CL07. Values are presented as mean ± standard error

Figure 11 shows the developmental stages of *A. salina* during exposure to different treatments. Based on these results, it was possible to verify development delays and arrest in some groups. For example, after 24 h of exposure, the microcrustaceans in the DB2 and DB10 treatments were unable to fully break the cyst wall and remained stalled at phase B (31.25% and 26.25%, respectively). In contrast, in the MM and MMS treatments, development proceeded only up to phase C, with 2.5% and 1.25% of individuals, respectively. The NC (55%), Aa (58%), PDAa (51.25%), and PDAf (51.25%) groups completed development up to instar III (Fig. 11F), with values above 50%, after 48 h.

### Acute lethality Assay

**Fig. 12:**
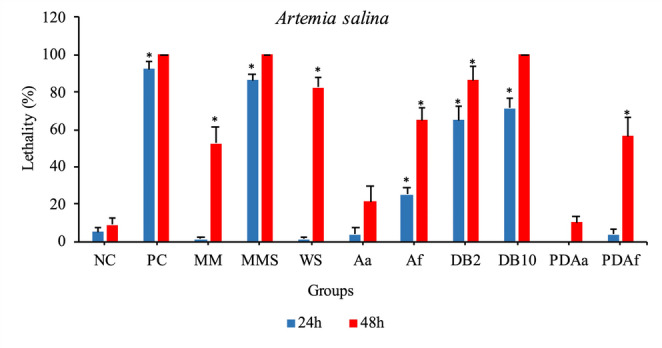
Lethality of *Artemia salina* over time (24 and 48 h) and treatment conditions. NC: Negative Control; PC: Positive Control; MM: Minimal Medium; MMS: Minimal Medium and Solvent; WS: Water and Solvent; Aa: *A. alternata* PL75; Af: *A. flavus* CL07; DB2: DB291 dye 2 µg/mL; DB10: DB291 dye 10 µg/mL; PDAa: Fungal decolorization product of *A. alternata* PL75; PDAf: Fungal decolorization product of *A. flavus* CL07. Bars represent the average number of deaths and the respective standard error *Statistically significant difference compared to the negative control (p˂0.05)

Figure 12 presents the results of the acute lethality of *A. salina* at 24 and 48 h. In the 24-hour period, the MMS, DB2, and DB10 groups significantly differed from the negative control, showing high lethality with percentages of 86.25%, 65% and 71.25%, respectively, approaching the lethality observed in the PC (90%). After 48 h of exposure, the PDAa treatment showed lethality similar to that of the NC group, with no statistically significant difference. In the other treatments, lethality increased over time, indicating that these treatments have an effect that becomes more pronounced as exposure progresses, although these effects were slower compared to the PC, MMS, DB2, and DB10 treatments, as they already exhibited lethality above 60% within 24 h. It is important to highlight that in the PC, MMS, and DB10 treatments, lethality reached 100% by the end of the exposure. Based on these data, it was observed that lethality was influenced by the dye concentration, treatment, and exposure time.

**Fig. 13:**
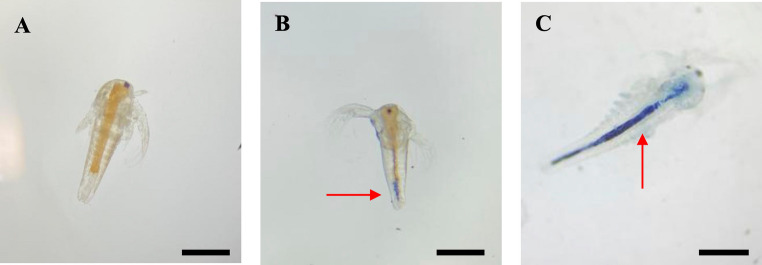
Nauplii after 48 h of exposure to DB291 dye. A: exposure to saline solution; B: exposure to saline solution and DB291 dye 2 µg/mL; C: exposure to saline solution and DB291 dye 10 µg/mL. Scale bar: 200 μm

Notably, during the lethality evaluation, individuals from the DB2 and DB10 groups exhibited the characteristic blue color of the dye throughout their intestines, which was not observed in the NC or in the groups corresponding to the fungal decolorization products (Fig. 13).

## Discussion

### Decolorization of disperse blue 291 by endophytic fungi

The *Aspergillus* genus has shown great biotechnological potential, and several studies have used these fungi for the decolorization of dyes and textile effluents. In a study conducted by Polli et al. (2023), which assessed the decolorization of the dye Reactive Black 5 using the fungus *A. flavus* CL07, it achieved 95.1% decolorization at a concentration of 0.1 g/L in seven days, and this fungus was tolerant to all the concentrations tested. According to Esmaeili and Kalantari (2012), *A. flavus* was effective in removing the azo dye Reactive Red 198 from wastewater, achieving 99.11% decolorization at a concentration of 50 mg/L in 24 h. According to Chau et al. (2023), the live biomass of *A. flavus* (3 g) demonstrated a considerable activity in the decolorization (48%) of textile effluents. The authors attributed this result to the high concentration of dye in the effluents from that treatment plant. Meanwhile, in the research conducted by Qin et al. (2024), A. *flavus* A5p1 achieved 93.59% decolorization in 120 h of exposure to the azo dye Reactive Orange 16 at concentrations ranging from 50 to 300 mg/L. Therefore, these findings are consistent with the results presented here.

In another approach, the study conducted by Ali and Muhammad (2008) demonstrated that *Alternaria solani* was efficient in the decolorization (88.6%) of the Acid Violet 19 dye at a concentration of 30 mg/L in 96 h. Chakraborty et al. (2013) reported that *Alternaria alternata* CMERI F6 achieved 99.99% decolorization of Congo Red dye at a concentration of 600 mg/L in 48 h. Although, in our study, *A. alternata* PL75 showed its efficiency (100% decolorization) in 168 h, the data reported above support the findings of the present research, highlighting the high efficiency of these fungi and the rapid decolorization performance of *A. flavus* CL07 in azo dye decolorization.

The decolorization pattern observed for *A. flavus* CL07 can be attributed to the unavailability of substrate in the medium, as it had already reached 100% decolorization, and to the abundance of secondary metabolites produced by this fungus, along with the products resulting from the breakdown of the dye DB291. This idea is supported by the study of Qin et al. (2024), who, while working with *A. flavus*, recorded a gradual decrease in the decolorization rate after 72 h at all studied concentrations. The authors attributed this finding to the toxicity of the Reactive Orange 16 dye, as well as to potentially toxic intermediate products, such as naphthalene and benzene compounds, produced during its degradation process. Pisoschi et al. (2023) state that the *Aspergillus* genus produces aflatoxins as secondary metabolites, which are characterized by their high toxicity.

The adsorption of dyes to fungal biomass constitutes the first mechanism of decolorization (Knapp et al. 1995; Syafiuddina and Fulazzaky 2021). According to Young and Yu (1997), the binding of dyes to the fungal hyphae, along with adsorption and degradation by extracellular enzymes, are the main mechanisms for color removal. Wang and Yu (1998) reported the adsorption of Acid Green 27, Acid Violet 7, and Indigo Carmine dyes onto both living and dead mycelia of the fungus *Trametes versicolor*. Jadhav and Govindwar (2006) stated that the retention of color in cells after dye adsorption indicates that decolorization occurs primarily as a result of biosorption. Tan et al. (2016) observed that the suspension and colonies formed during the process were colored after the decolorization of Reactive Green KE-4BD dye, suggesting that part of the effluent was decolorized by dye adsorption. These findings reinforce the hypothesis that the dye was adsorbed by these fungi.

Other studies demonstrate ongoing advancesin the development of methodologies for treating these contaminants, such as the use of nanobiocomposites, which have recently shown significant potential. El-Sharkawy et al. (2024) synthesized zinc phosphate nanosheets using exometabolites from *Aspergillus versicolor* incorporated into alginate microspheres. This material achieved up to 90% biosorption of methyl orange even after six reuse cycles. Similarly, El-Sharkawy and Abbas (2023) utilized extracellular secretions from *Aspergillus fumigatus* to produce zinc phosphate-based nanoparticles for the adsorption of methylene blue and methyl orange. The maximum adsorption capacities (q_max_) were 178.25 mg/g and 50.10 mg/g, respectively, with the regenerated material maintaining high efficiency after successive cycles. These findings, alongside the results of the present study, highlight sustainable and cost-effective biotechnological approaches that can be effectively applied to the bioremediation of environments contaminated by textile dyes.

### Enzymatic degradation of disperse blue 291

Qin et al. (2024) state that the degradation of xenobiotics by microorganisms is primarily carried out with the involvement of enzymes synthesized and secreted by the cells. This process can be either extracellular or intracellular. Thakkar and Bhatt (2020) mention that aromatic compounds induce the production of laccase. In the present study, it was observed that the DB291 dye significantly stimulated the production of extracellular enzymes, particularly laccase, for both fungi, but with a higher level for the *A. alternata* PL75 strain.

The enzymatic patterns observed for *A. flavus* CL07 were also reported by Qin et al. (2024). Additionally, Irfan et al. (2018) suggest that the decrease in laccase activity may be associated with nutrient depletion in the medium. However, according to Alam et al. (2023), the longer the decolorization process, the greater the secretion of laccase, which corroborates the results of the present study.

Research has highlighted the production of the enzyme laccase by fungi from the *Aspergillus* and *Alternaria* genera. Additionally, studies have demonstrated that laccase is among the enzymes capable of degrading textile dyes, as it reacts with various aromatic compounds, such as azo compounds, thereby showing its effectiveness in decolorization (Jasinska et al. 2012; Giovanella et al. 2020; Qin et al. 2024). Since laccase was identified in the analysis of the decolorization product, this suggests its extracellular nature (Jasinska et al. 2012).

The results indicated that both laccases and peroxidases-like activity played a role in the decolorization process. It is important to note that while ABTS is a standard substrate for assessing ligninolytic enzymes, the activity measured here represents the cumulative oxidative potential of the fungal secretome. Although the subtraction method is a common screening tool, other oxidoreductive enzymes beyond laccases and peroxidases may contribute to the total oxidation of the substrate. This cumulative action may suggests a synergistic effect in the breakdown of the azo dye. However, more in-depth studies need to be conducted to characterize all the enzymes involved in decolorization, particularly for *A. alternata* PL75, as there are few studies focusing on this genus in relation to decolorization.

### Possible dye degradation pathways

The decolorization and degradation of azo dyes initially occur through the cleavage of the azo group’s double bond (Chang et al. 2008; Qin et al. 2024). The decolorization pathways of these dyes are complex, influenced by their spatial arrangement, the physiological metabolism of fungi, and qualitative and quantitative differences in the enzymes they produce (Chen et al. 2021; Qin et al. 2024).

Quantification of the dye in the mycelium after decolorization indicated that, for *A. flavus* CL07, the entire dye was adsorbed onto the mycelium (biosorption). In contrast, for *A. alternata* PL75, biosorption occurred alongside partial biodegradation of the dye.

FTIR-ATR analysis revealed the disappearance of certain spectral bands post-decolorization, suggesting that the dye induces structural modifications or degradation of microbial proteins, nucleic acids, and phospholipids, potentially resulting in the formation of products that interact with DNA. Umbuzeiro et al. (2005), using the Ames test, demonstrated that the dye DB291 caused frameshift mutations (hisD3052) and all types of base substitutions with a preference for TA over AT, as well as CG to TA and CG to AT transitions. Tsuboy et al. (2007) further reported that DB291 exhibits genotoxic and mutagenic effects causing DNA fragmentation, micronuclei formation, and increased apoptosis in mammalian cells.

The observed shift in the N–H amide and O–H carboxylic acid spectral regions reflects the interaction of the dye with fungal functional groups such as N–H and O–H, which may lead to a reorganization of molecular structures. This supports the hypothesis that the dye undergoes biosorption or degradation by the fungus (Singh and Dwivedi 2020).

Moreover, the emergence of new bands may indicate the formation of novel chemical groups or molecular configurations resulting from dye degradation or modification during the decolorization process. Similar observations have been reported by Lade et al. (2012) and Kumar and Dwivedi (2019). Other studies have noted comparable trends in synthetic dye decolorization and emphasized that dye biosorption is influenced by heteropolysaccharides and lipid components of the microbial cell wall, which contain various charged functional groups such as hydroxyl, carboxyl, and phosphate groups, generating strong attractive forces within the cell wall (Chakraborty et al. 2013; Almeida and Corso 2014; Mota et al. 2015; Rodrigues de Almeida et al. 2019; Polli et al. 2023).

The spectral shift observed in the *A. alternata* PL75 spectrum after decolorization (3700–3000 cm⁻¹) may indicate significant alterations in molecular structure and chemical interactions, suggesting increased bond strength, including stronger hydrogen bonds or electrostatic interactions, that stabilize the molecules (Singh and Dwivedi 2020). The two newly appearing low-intensity bands (900–700 cm⁻¹), with peaks characteristic of unsubstituted and multisubstituted aromatic rings such as naphthalenes or benzenes, correspond to bending vibrations of C–C and stretching of C–H bonds in substituted aromatic compounds. Following decolorization, the increased intensity in this region implies structural changes in the dye’s aromatic ring. Notably, this spectral region disappears in the mycelium spectrum after decolorization, suggesting that the fungus can degrade or extensively modify the aromatic rings associated with these vibrations (Iark et al. 2019), or alternatively, adjust its metabolic pathways.

Overall, these results indicate that *A. flavus* CL07 primarily employs biosorption as the initial mechanism, as UV-Vis quantification showed that all dye used in the assay was recovered in the mycelium at the same concentration. Conversely, *A. alternata* PL75 likely performs both biosorption and enzymatic biodegradation via extracellular enzymes it produces. According to Singh and Dwivedi (2020), the disappearance or appearance of new FTIR peaks provides evidence of the biotransformation of azo dyes into various metabolites.

### Ecotoxicology assessment

Regarding the development of *A. salina*, Sorgeloos et al. (1978) and Carballo et al. (2002) stated that this microcrustacean is highly vulnerable to toxic substances during the early stages of development. Alyürük and Çavaş (2013), analyzing the hatching ability of *Artemia* in response to the pesticide diuron (25 mg/L), found that the organisms were unable to fully rupture the cyst wall, resulting in a halt in their development. A study conducted with palytoxin, a highly toxic polyether found in some marine animals, significantly reduced cyst hatching (Cavion et al. 2022). Rotini et al. (2015) investigated the toxicity of diethylene glycol and sodium dodecyl sulfate on *Artemia franciscana*. According to these authors, hatching inhibition was dose-dependent. These findings indicate that toxic substances can inhibit or delay the hatching of *Artemia* cysts, which supports the results presented in this study.

Notably, the results obtained for the fungus *Aa* and, especially, for the decolorization products of both fungi (PDAa and PDAf), showed hatching rates above 50%. It was also observed that the hatching success in the PDAa treatment was 16% higher than in the PDAf treatment. These findings highlight the potential of *A. alternata* in the bioremediation of this textile dye. The *Aspergillus* genus is known for its aflatoxicity; however, the strain used in this study, *A. flavus* CL07, although non-aflatoxigenic (Balbinot et al. 2021), may still produce other toxins also reported for this genus (Polli et al. 2023).

Studies by Ayed et al. (2011) observed lower lethality in the decolorization product of the azo dye Methyl Green compared to the raw dye (750 mg/L). Pizato et al. (2017), analyzing the toxicity of untreated industrial effluent and its treatment with the fungus *Lasiodiplodia theobromae*, demonstrated that the raw effluent concentration caused 50% mortality in *A. salina*. According to Bilal et al. (2016), the azo dye Reactive Black 5 led to complete mortality in *A. salina*, whereas, after treatment of the dye (UV photoirradiation in an aqueous titanium dioxide suspension), the degraded metabolites induced a low mortality rate, highlighting the less toxic nature of the degraded metabolites compared to the untreated dye. These findings align with the results of the present study.

Another important point to highlight is that the dye at a concentration of 2 µg/mL, the amount present in the decolorization products of the fungi studied, exhibited considerable lethality (86.25% at 48 h). However, this result raises concern due to the hazardous nature of azo dyes, but it is expected, as dispersed dyes are known to be hydrophobic and can easily adsorb onto aquatic sediments or form stable suspensions that can be transported by receiving waters and reach water-treatment plants (Vacchi et al. 2017; Fernandes et al. 2019). Therefore, this characteristic of the DB291 dye can help explain its highly toxic nature even at low concentrations.

*Artemia* is a genus of non-selective filter-feeding microcrustaceans that ingest water and particles available in the environment (Wang et al. 2019; Charoeythornkhajhornchai et al. 2023), and in marine organisms, the intestine is a target organ, especially for zooplankton (Jemec et al. 2016; Wang et al. 2019). Croghan (1958), studying the swallowing behavior of *Artemia* in hypertonic, isotonic, and hypotonic saline environments, found that the animal actively ingested a medium composed of a phenol red solution, and within a few hours, the entire intestinal lumen turned red. The author believes that *Artemia* possesses a mechanism in its intestinal epithelium for the active absorption of water. The findings presented in this study support these results.

A scarcity of literature addressing the bioaccumulation of textile dyes in *Artemia* has been identified. Consequently, ecotoxicological studies involving aquatic bioindicators, such as platyhelminths (freshwater planarians) and fish, were examined. Despite the differences in target organs and toxic mechanisms among these organisms, the objective was to gain insight into the potential effects of the dye on these bioindicator models.

Ribeiro and Umbuzeiro (2014) studied the sensitivity of the planarian *Girardia tigrina* to the textile dye Disperse Red 1. After 24 h of exposure in acute toxicity tests, both newborn and adult planarians exposed to concentrations greater than 10 mg/L exhibited several alterations, including reddening of the skin, particularly in the cephalic region. The authors explained this as a result of dye adsorption onto the skin or cilia during locomotion and/or skin respiration. Leite et al. (2024) studied acute toxicity in *Danio rerio* (zebrafish) embryos exposed to different dyes, and found that Disperse Red 60 caused ocular alterations in the embryos. This raised environmental concerns for the authors, as it impacts the animal’s physiological processes, such as food seeking and mating behavior, and makes it more vulnerable to predators. On the other hand, this issue poses an imminent risk to human health, since the ocular system of zebrafish resembles the human visual system in terms of development, size, and morphology. Additionally, the mutagenic effects of some textile dyes have already been reported in the literature using other test systems (Umbuzeiro et al. 2005; Caritá and Marin-Morales 2008; Franco et al. 2018).

However, the results presented in this study raise concern, as the bioindicator *A. salina*, according to Migliore et al. (1997) and Alvarez-Alarcón et al. (2021) has a great osmoregulatory capacity, which contributes to its high resistance to toxic substances. Nonetheless, in this study, the DB291 dye exhibited total lethality (100%) at the highest concentration tested (10 µg/mL). Therefore, studies of this nature, which focus on the treatment of xenobiotics with endophytic microorganisms and their ecotoxicological evaluation, are becoming increasingly necessary, as the metabolism of these products may generate compounds that are increasingly hazardous to human health and the environment.

On the other hand, industrial production is continually expanding, seeking raw materials that are more resistant to abiotic factors, with greater chemical stability and a long half-life. This, to some extent, may increase the likelihood that pollutants persist in the environment. Therefore, it becomes essential to continuously test these products before they are released to the market to ensure greater safety for the population and ecosystems. However, this study emphasizes the importance of the biological treatment of dyes and textile effluents, as well as their ecotoxicological evaluation, for environmental sustainability, which represents significant potential for the bioremediation of contaminated environments.

Despite the promising results in DB291 dye decolorization and ecotoxicity reduction, this study has some limitations. First, the assays were conducted at a laboratory scale under controlled culture media and agitation conditions, which may not fully reflect the complexity and variations in pH and temperature of real textile effluents. Furthermore, although *Artemia salina* is an excellent bioindicator for initial screening, the environmental safety assessment of the bioremediation process would benefit from additional tests involving other trophic levels, such as algae or fish. Lastly, the detailed identification of all intermediate metabolites resulting from fungal degradation still requires more in-depth analysis using techniques like mass spectrometry to fully elucidate the metabolic pathways involved.

In conclusion, azo dyes are a group of pollutants that raise concerns regarding the environment and ecological balance. However, endophytic fungi have garnered significant interest in the scientific community for the biodegradation of these dyes, due to their biotechnological potential and ability to adapt their metabolic pathways when exposed to pollutants. This study demonstrated the efficiency of two fungi in the decolorization of the textile azo dye Disperse Blue 291. The results showed that the fungus *A. flavus* CL07 exhibited rapid decolorization within 48 h, while *A. alternata* PL75, although reaching complete decolorization at 168 h, proved to be more effective, as it not only biosorbed the dye but also biodegraded it, with the extracellular enzyme laccase likely involved in this mechanism. The ecotoxicological analysis revealed the toxicity of the dye at the concentrations analyzed, causing delays or arrest in the development of *A. salina*, which resulted in high mortality rates. In contrast, the decolorization product from the fungus *A. alternata* significantly reduced the toxicity of the dye DB291. These findings highlight the potential application of these fungi in the biosorption/biodegradation of the azo dye DB291 and provide further support for the use of these fungi in the bioremediation of contaminated environments.

## Data Availability

The datasets generated during and/or analyzed during the current study are available from the corresponding author on reasonable request.

## Abbreviations

Aa: *Alternaria alternata* PL75
ABTS: 2,2-azino-bis(3-ethylbenzothiazoline-6-sulfonic acid)
Af: *Aspergillus flavus* CL07
ATR: Attenuated Total Reflectance
C.I.: color index
CMEA: Collection of Endophytic Microorganisms and the Environment
DB10: Disperse Blue 291 10 µg/mL
DB2: Disperse Blue 291 2 µg/mL
DB291: Disperse Blue 291
DMSO: dimethyl sulfoxide
FTIR: Fourier Transform Infrared Spectroscopy
MM: Minimal Culture Medium
MMS: Minimal Medium and Solvent
NC: Negative Control
PBTA-7: phenylbenzotriazole
PC: Positive Control
PDA: Potato Dextrose Agar
PDAa: Decolorization product of Alternaria alternata PL75 + DB291 10 µg/mL
PDAf: Decolorization product of *Aspergillus flavus* CL07 + DB291 10 µg/mL
SisGen: National System for the Management of Genetic Heritage and Associated Traditional Knowledge (Brazil)
UV-Vis: ultraviolet-visible
V_d_: Decolorization Rate
WS: Water and Solvent
